# Identification of protein complexes that bind to histone H3 combinatorial modifications using super-SILAC and weighted correlation network analysis

**DOI:** 10.1093/nar/gku1350

**Published:** 2015-01-20

**Authors:** Natalia Kunowska, Maxime Rotival, Lu Yu, Jyoti Choudhary, Niall Dillon

**Affiliations:** 1 Gene Regulation and Chromatin Group, MRC Clinical Sciences Centre, Imperial College, Hammersmith Hospital Campus, Du Cane Road, London W12 0NN, UK; 2 Integrative Genomics and Medicine Group, MRC Clinical Sciences Centre, Imperial College, Hammersmith Hospital Campus, Du Cane Road, London W12 0NN, UK; 3 Proteomic Mass Spectrometry, Wellcome Trust Sanger Institute, Cambridge, CB10 1SA, UK

## Abstract

The large number of chemical modifications that are found on the histone proteins of eukaryotic cells form multiple complex combinations, which can act as recognition signals for reader proteins. We have used peptide capture in conjunction with super-SILAC quantification to carry out an unbiased high-throughput analysis of the composition of protein complexes that bind to histone H3K9/S10 and H3K27/S28 methyl-phospho modifications. The accurate quantification allowed us to perform Weighted correlation network analysis (WGCNA) to obtain a systems-level view of the histone H3 histone tail interactome. The analysis reveals the underlying modularity of the histone reader network with members of nuclear complexes exhibiting very similar binding signatures, which suggests that many proteins bind to histones as part of pre-organized complexes. Our results identify a novel complex that binds to the double H3K9me3/S10ph modification, which includes Atrx, Daxx and members of the FACT complex. The super-SILAC approach allows comparison of binding to multiple peptides with different combinations of modifications and the resolution of the WGCNA analysis is enhanced by maximizing the number of combinations that are compared. This makes it a useful approach for assessing the effects of changes in histone modification combinations on the composition and function of bound complexes.

## INTRODUCTION

Whole genome sequencing has provided unprecedented information in recent years about gene structure and organization but it is also clear that many aspects of gene regulation are controlled epigenetically by chromatin. Multiple signalling pathways converge on the core histones, which are subject to extensive post-translational modification. The N-terminal tails of the histones, which extend out from the nucleosome core, have a particularly high density of covalent modifications that include acetylation, methylation, phosphorylation, ADP-ribosylation, ubiquitination and sumoylation. One of the major functions of these post-translational modifications is to act as docking sites for binding of chromatin proteins. Binding of these ‘reader’ proteins to the histone tails creates a focal point for recruitment of chromatin-modifying complexes that mediate changes to the higher order structure of chromatin and binding of transcriptional activators or repressors. Recognition of histone marks by their readers is therefore a crucial step in translating epigenetic modifications into meaningful biological outcomes. However, deciphering the functions of histone modifications requires much more than matching single histone marks with their binding partners. The majority of histone modifications do not work in isolation. On the contrary, they form a combinatorial histone code or language, with some modifications having the potential to affect the recognition and binding of specific readers to modifications at other residues, either antagonistically or agonistically. The number of combinations of histone modifications that are used is considerably less than the vast number that could potentially exist, but there is good evidence of a wide range of effects of different combinations generated by multivalent binding of histone reader proteins, by the presence of proteins with different histone modification specificities in the same reader complex and by allosteric effects of histone binding on protein function (reviewed in (1)). When the complexity of large protein complexes that bind to histones is also considered, the potential for very subtle biological effects is clear. In this study, we set out to use systematic high-throughput approaches to study the dynamics and composition of protein complexes that bind to combinations of histone modifications. As a model system, we chose to study the complexes that bind to the combinatorial modifications that are generated on the histone H3 tail by the presence of lysine (K) residues immediately adjacent to serine (S). The amino-terminal tail region of histone H3 (residues 1–30) contains two such combinations that involve the key histone H3K9 and K27 residues, which are known to be involved in chromatin-mediated repression. H3K9me3 binds members of the heterochromatin protein 1 (HP1) family and is a canonical marker for heterochromatin. H3K27me3 plays a key role in maintaining ES cell pluripotency and regulating cell differentiation by acting as a recognition signal for the repressive polycomb complexes PRC1 and PRC2. H3K9 and H3K27 are also targets for acetylation. H3K9ac is found at many active promoters and H3K27ac is a marker for enhancer activity. The fact that K9 and K27 are both located next to serine residues (S10 and S28) has the potential to generate phospho-methyl and phospho-acetyl switches. Double H3K9me3/S10ph and H3K27me3/S28ph modifications have been detected using antibodies that specifically recognize the combined modifications and have been shown to be present at different stages of the cell cycle and to be involved in regulating protein binding during cell differentiation (2,3). Phosphorylation of H3S10 or S28 has been shown to affect binding of HP1 proteins to H3K9me3 and binding of polycomb proteins to H3K27me3.

We have used peptide capture in conjunction with a super-SILAC-type approach (4) to search for protein readers that bind to H3K9me3, H3K9me3/S10ph, H3K27me3 and H3K27me3/S28ph. The method allows us to accurately quantify binding and directly compare levels of binding to peptides that carry a number of different methyl-phospho combinations. This makes it possible to use weighted correlation network analysis (WGCNA) (5,6) as an unbiased method for grouping factors together based on their binding ‘signatures’ and to use these signatures to identify complexes that recognize specific combinations of modifications. Our results provide evidence that proteins bind to histone modifications as pre-assembled complexes with combinations of modifications affecting the composition of bound complexes. We identify complexes that bind to H3K9me3 and S10ph as well as a novel complex binding to the double K9me3/S10ph mark. Our data show that formation of the double H3K9me3/S10ph modification acts as a variable switch that displaces binding of many proteins but leaves others unaffected or showing enhanced binding.

## MATERIALS AND METHODS

### Cell culture, SILAC labelling and transfection

Murine myeloma MPC11 cells were grown in SILAC DMEM (Pierce), 10% dialyzed horse serum (Biosera) with the addition of either 100 μg/ml lysine and arginine or heavy-labelled lysine-8 and arginine-10 (CK Gas Products Ltd) for at least five cell divisions. For western blot experiments, HEK293 cells were grown in DMEM/10% fetal bovine serum (FBS) (Gibco) and transfected using the PEI method (7). The cells were harvested 24 h after transfection and nuclear and chromatin extracts were prepared.

### Extract preparation

Cells were harvested by centrifugation, washed once with ice-cold phosphate buffered saline and resuspended in 1 ml sucrose buffer (10 mM Tris pH 8.0, 0.32 M sucrose, 3 mM CaCl2, 2 mM magnesium acetate, 1 mM dithiothreitol (DTT), 0.1 mM ethylenediaminetetraacetic acid (EDTA), 0.5% NP-40 and freshly added protease inhibitors (Roche)) per 10^8^ cells to isolate the nuclei. After 5 min of incubation on ice, the nuclei were pelleted by centrifugation (500 g, 5 min, 4°C) and washed once with sucrose buffer without the detergent. The quality of the nuclear preparations was confirmed visually by trypan blue staining. The nuclei were then resuspended in 1 ml lysis buffer (20 mM Hepes, pH 7.9, 25% v/v glycerol, 420 mM KCl, 1.5 mM MgCl2, 0.2 mM EDTA, 1 mM DTT and freshly added protease inhibitors) per 3 × 10^8^ cells and subjected to three cycles of freezing and thawing, followed by centrifugation (10 min, max 4°C). The supernatant was collected as the nuclear fraction, while chromatin pellets were solubilized by digestion with MNase I (New England BioLabs). The nuclear and chromatin fractions were pooled together and used for the peptide capture or immunoprecipitation (IP) assays.

### Peptide design

Peptides derived from the N-terminus of histone H3, corresponding to either aa1–20 or 18–38 (peptide 1 or 2, respectively) were synthesized by GL biochem. A biotin moiety that allowed for coupling to Avidin beads (NeutrAvidin, Pierce) was attached to the C-termini of the peptides via a glycine-lysine linker. The peptides were either unmodified or contained the following defined post-translational modifications: trimethylation of lysine K9 (peptide 1) or K27 (peptide 2), phosphorylation of serine S10 (peptide 1) or S28 (peptide 2) or the two modifications combined. Beads alone were used as the negative control.

### Peptide pulldown

The peptide capture assay was performed as described (8) with the following modifications. For SILAC experiments, 10^8^ cells were used per assay. For each assay, three independent extractions were performed and the extracts were pooled together. Extracts adjusted to 150 mM NaCl and pre-cleared were incubated at 4°C with 20 μl of NeutrAvidin beads (Pierce) coupled to biotinylated histone peptides. Beads alone were used to control for non-specific binding. After the incubation, the beads were washed five times with ice-cold wash buffer (20 mM Hepes, pH 7.9, 20% v/v glycerol, 0.2 mM EDTA, 0.2% Triton X-100, 150 mM KCl and freshly added protease inhibitors). Bound proteins were eluted by boiling in Laemmli buffer. Heavy and light pulldown samples were mixed as indicated, then run on 10% sodium dodecyl sulphate-polyacrylamide gel electrophoresis (SDS-PAGE) gel and subjected to mass spectrometry analysis. For quantitative western blot analysis, extracts from 0.1–0.5 × 10^8^ cells per peptide capture assay were used. Eluted proteins were subjected to SDS-PAGE followed by western blotting and detection with the indicated antibodies. The western blots were visualized using ImageQuant LAS 4000 mini (GE Healthcare) and quantified with Multi Gauge software (FUJI FILM).

### Immunoprecipitation

For IP experiments, 5 μg of antibody were pre-coupled to 50 μl of protein G Dynabeads (Invitrogen) and the IP reaction was carried out for 4 h at 4°C. Beads were washed three times in wash buffer (20 mM HEPES, pH 7.9, 150 mM NaCl, 1.5 mM MgCl2, 0.2 mM EGTA, 0.2% Triton X‐100 and 10% glycerol). Bound proteins were eluted by boiling in Laemmli buffer. Heavy specific pulldowns were mixed with the corresponding light isotope controls, and then run on 10% SDS-PAGE gels and subjected to mass spectrometry analysis. The heavy-to-light ratios (H/L) of co-immunoprecipitated proteins were normalized against the corresponding H/L ratio obtained for pulldown of the bait protein. The values obtained were used to approximate the strength of interactions for the construction of the ‘saddlebrown’ module interaction network in Cytoscape v. 3.1.0 (9), where they are represented as the weight of the edges.

### Mass spectrometry

Each gel lane was excised into three or five equal pieces, which were destained with 50% 100 mM ammonium bicarbonate/50% acetonitrile (ACN). Proteins in the gels were reduced with 10 mM dithiothreitol, then alkylated with 55 mM iodoacetamide. Trypsin (20 ng) was added to each of the gel pieces followed by incubation overnight at 37°C. Peptide extraction was carried out in 5% formic acid (FA).

LC-MS analysis was on an LTQ Orbitrap Velos mass spectrometer (Thermo Fisher) coupled to an Ultimate 3000 RSLCnano LC system. Peptides were resuspended in 0.1% trifluoracetic acid (TFA) and were then loaded onto a 100 μm × 2 cm PepMap C18 trap (100 Å, 5 μm) separated on a 75 μm x 50 cm PepMap C18 column (100 Å, 2 μm) (both from Thermo Fisher) using a linear gradient of 4 to 55% B in 65 min (solvent A: 0.1% FA/98% H_2_O, 2% ACN, solvent B: 0.1%FA/80%, ACN/20%H_2_O). The instrument was controlled by the Xcalibur software with a standard CID top six data dependent acquisition method. The resolution of Full MS survey was set at 15 000. The parent ion's isolation width was set at 2.0 Da, and the normalized collision energy at 35.0, activation Q at 0.25, activation time 30 ms and the lock mass at 445.120030 m/z.

### Data processing

3D peak detection and quantification was performed by MaxQuant (v 1.3.0.5) and protein identification was performed by the embedded Andromeda search engine and the Uniprot mouse database (release May 2013) with default parameters: the peptide mass tolerance at first search was set at 20 ppm and main search at 6 ppm; MS/MS fragment mass tolerance at 0.50 Da and top 6 MS/MS peaks per 100 Da and a minimum peptide length of six amino acids were required. A maximum of three labelled amino acids, five modified amino acids and two missed cleavages of trypsin/P were allowed per peptide. Protein N-terminal acetylation, oxidation of methionine and deamindation of aspargine and glutamine were set as variable modifications and the carbamidomethyl on cysteine as a fixed modification. The false discovery rates (FDR) for both peptide and protein were set to 1% using a reversed database as the decoy database. The reported protein groups had to contain at least one razor peptide. The protein groups output table was filtered for common contaminants and identifications from the decoy database, as well as peptide length, mass error precision and peptide score. The re-quantification feature was enabled. The protein quantification used razor and unique peptides. A minimum of two ratio counts (redundant peptides used for quantification) was required. This resulted in a set of 1326 proteins, which were used as a starting point for further analyses in R.

### Weighted gene co-expression network analysis

The weighted correlation network was constructed using the freely accessible R software package as previously described (5,6). In detail: before the analysis, proteins with 15 or more missing values were filtered out. This resulted in a dataset of 1023 proteins, which had been quantified in at least four out of 18 peptide capture samples. We have arrived at this criterion by testing WGCNA network construction using proteins identified in at least 1, 2, 3 up to 9 out of the 18 pulldown samples. This approach showed that using proteins that had been quantified in at least four samples for WGCNA ensured that the resulting network is robust and enriched in proteins with high topological overlap while maximizing number of proteins used for the analysis (10). As our experimental set up included enrichment by affinity pulldown, the distribution of the H/L values was not expected to be normal. Therefore, we have used non-normalized H/L ratios for all analyses. For WGCNA, the missing values were imputed to zero. For the selected proteins, a pairwise correlation matrix across the 18 samples was calculated. Next, a soft power threshold β of 15 was used to transform the correlation matrix into a signed weighted adjacency matrix, leading to an approximate scale-free topology of the obtained network. The resulting adjacency matrix was then used for calculation of the topological overlap matrix (TOM), which is a robust measure of network interconnectedness. A cluster dendrogram, generated by hierarchical clustering of proteins on the basis of their topological overlap, was cut into modules of minimal size five using a Dynamic Tree Cut algorithm. Proteins that could not be assigned to a specific module were grouped in the ‘grey’ module. The binding profile of each module was summarized by its first principal component (the module eigenprotein). The hub proteins of each module were identified based on their intramodular connectivity (kWithin), which was calculated for each protein by summing the connection strengths (adjacencies) with other module proteins and dividing this number by the maximum intramodular connectivity in a given module. For a more detailed discussion on using on using WGCNA to study the interactome of combinatorial histone marks, see Supplementary Materials.

### STRING

The online STRING 9.1 database (http://string.embl.de/) was then used to identify previously described interactions within the WGCNA modules. The input options were set to include ‘Co-occurrence’, ‘Co-expression’, ‘Experiments’, ‘Databases’ and ‘Textmining’ with a confidence level of 0.4. We have used the in-built STRING functions to test each module for enrichment in interactions and specific Gene Ontology (GO) terms. The constructed networks were then exported as text files to Cytoscape v. 3.1.0 (9). The combined score for each interaction was represented as the width of the edge between the two nodes, while the node size corresponded to the kWithin value from WGCNA.

### Statistical analyses

For testing the statistical significance of the enrichment of each module in a specific pulldown versus ‘beads-only’ negative control, we considered enrichment values for all of the proteins in the module simultaneously. For each specific pulldown condition, a paired two-tailed *t*-test was used to assess whether binding of proteins belonging to the same module was significantly higher than the binding observed on beads alone. Supplementary Table S1 contains the calculated *p*-values.

For the outlier analysis for each pulldown, the mean H/L ratio was calculated from the two replicates, ignoring the missing values. Next, the mean values were log_2_ transformed and for each protein the negative control value representing binding in the beads-only pulldown was subtracted from the values obtained for the pulldowns with specific peptides. To test whether a particular protein ratio is a significant outlier with respect to the distribution of all other proteins in the pulldown we calculated the *z*-score with the assumption of no enrichment in binding over beads. In this setup, the *z*-score would correspond to the number of standard deviations above zero. From the *z*-scores, we have then calculated the *p*-value using a normal distribution for each protein in the pulldown. The obtained *z*-scores and *p*-values are summarized in Supplementary Table S1.

## RESULTS

### Analysis of protein binding to histone H3 methyl-phospho modifications

**Figure 1.**
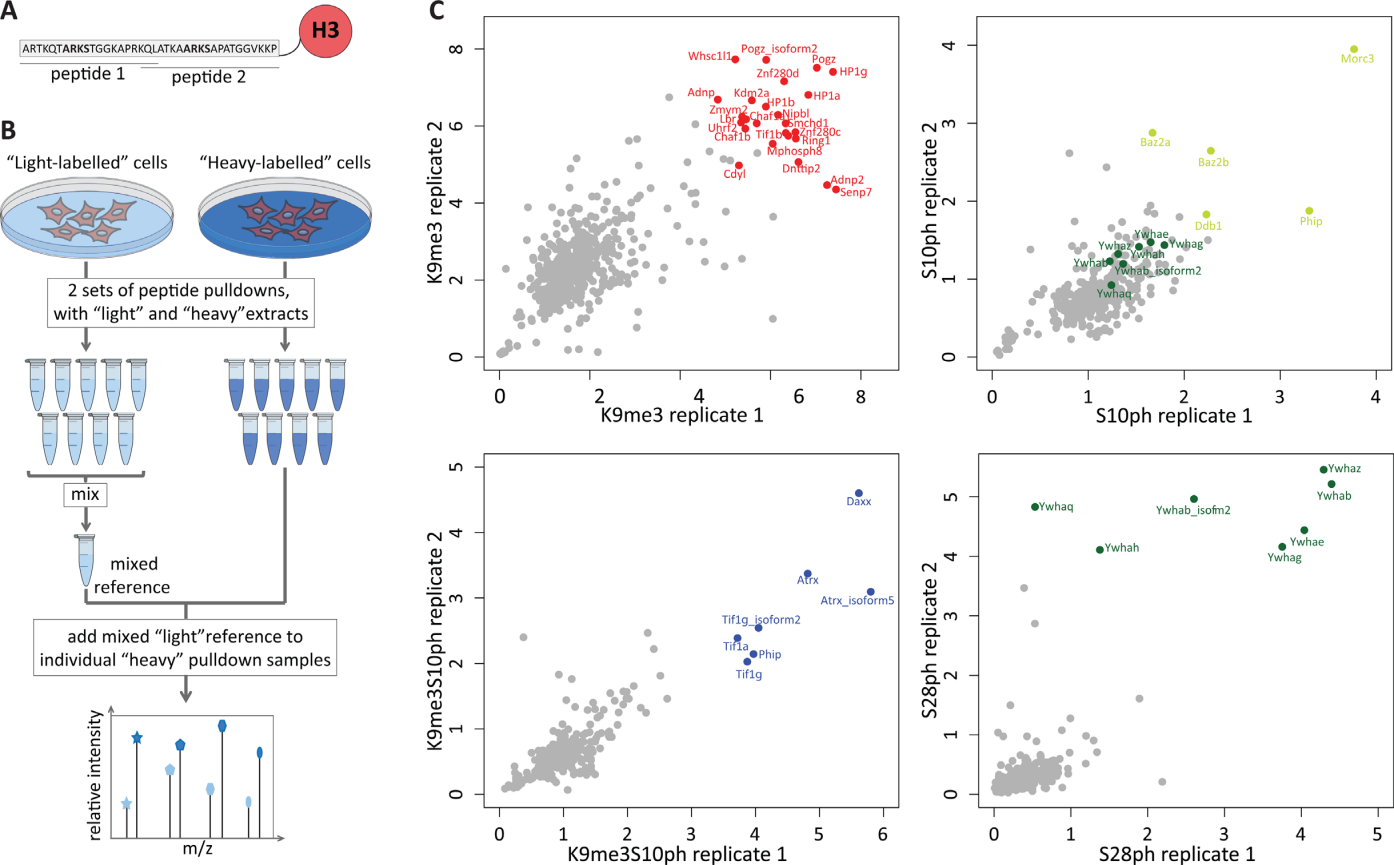
Mixed reference used in comparative quantitative proteomic identification of proteins reading combinatorial histone marks. (**A**) Peptide design for peptide capture assays. Two peptides derived from the H3 tail, encompassing amino acids 1–20 and 18–20, were used. The ARKS motifs which are the targets for methylation and phosphorylation are marked in bold. (**B**) Experimental design. MPC11 cells were grown in ‘light’ and ‘heavy’ SILAC media. Nuclear extracts from each condition were used for nine pulldowns (eight peptide capture samples and one ‘bead-only’ negative control). The ‘light’ set was then mixed, divided into nine equal parts and spiked into the ‘heavy’ samples as a common reference for mass spectrometry quantification. Samples were then subjected to LC-MS/MS. (**C**) Scatter plots of replicate 1 versus replicate 2 for H3K9me3 pulldown (top left), H3S10ph (top right), H3K9me3S10ph (bottom left) and H3S28ph (bottom right). The x and y axes represent the H/L ratios that correspond to enrichment over the mixed reference. The top binders for each peptide have been highlighted in red (for K9me3), blue (K9me3S10ph), light green (S10ph) and dark green (S28ph). Additionally, for H3S10ph 14-3-3 proteins, the putative readers of the S10 mark are highlighted in dark green to distinguish them from the other H3S10ph binders. For binding to H3S28ph, five of the seven 14-3-3 proteins show a good correlation in both replicates. Two 14-3-3 proteins (Ywaq and Ywah) showed a lower enrichment in replicate 1 (bottom right panel). This experimental variation could reflect a less stable interaction of these family members with the histone tail.

A peptide capture assay in conjunction with SILAC analysis was used to compare binding profiles of nuclear proteins from MPC11 myeloma cells to peptides carrying the H3K9me3, H3K27me3, H3S10ph, H3S28ph and the double H3K9me3/S10ph and H3K27me3/S28ph modifications. The analysis was carried out using peptides corresponding to amino acids 1–20 and 18–38 and the unmodified peptides were also analysed to assess binding to the unmodified histone tail (Figure 1A). In principle, it is possible to directly compare binding up to a maximum of three modified peptides by carrying out pulldowns with ‘light’, ‘medium’ and ‘heavy’ extracts. However, this number would be insufficient to allow direct comparison of the binding profiles for the eight combinations described above. An alternative strategy would be to compare pull-downs between each modified peptide with a common unmodified peptide that serves as the internal control. The limitation of this approach is that for proteins that show little or no binding to the unmodified peptide, this results in division by very small numbers (background), which can lead to large errors in the estimation of levels of binding to different modifications (Supplementary Figure S1).

In order to overcome this problem and ensure that the proteins present in any of the capture assay samples have corresponding peptides in the common internal reference, we adapted the super-SILAC approach (4) using a reference sample that was obtained by mixing the eight pulldowns and the negative beads-only control (Figure 1B). Briefly, the MPC11 cells were grown in media containing light- and heavy-labelled arginine and lysine. Nuclear extracts from these cells were then incubated with the eight peptides harbouring the defined modifications conjugated to Neutravidin beads and with the beads alone as the negative control. After the elution, the ‘light’ set of pulldowns was pooled together creating a reference mix, which was then divided into nine equal aliquots. An aliquot of the reference mix was added to each separate ‘heavy’ peptide pulldown reaction. The samples were then processed and subjected to mass spectrometry to identify and quantify the captured proteins. The ratios obtained correspond to the enrichment of a given protein in the individual pulldown sample versus the mixed reference (Figure 1B). Since our experimental set up consisted of eight peptide capture samples plus one negative control, the predicted sum of H/L ratios for a given protein equals nine. Indeed, for the subset of proteins that had no missing values, 85% of the summed ratios fall between 7 and 10, approximating the theoretical prediction (Supplementary Figure S2A).

The experiment was performed in two replicates, which showed a good correlation (Figure 1C, Supplementary Figure S2B and Supplementary Table S1). In the H3K9me3 capture assays, the HP1 family members and known HP1-associated proteins (Zmym3, Tif1β, Znf828, Pogz, Znf280d, Nipbl, H3K9-trimethyltransferase Suv39h and the histone chaperones Chaf1a and Chaf1b) (11,12) were among the most highly enriched proteins in both replicates, further confirming the quality of the data (Figure 1C, top left, red). The 14-3-3 proteins were very strongly enriched in the H3S28ph pulldown (Figure 1C, bottom right, dark green) and to a lesser degree in the pulldown with the S10-phoshorylated H3 peptide (Figure 1C, top right, dark green). This correlates well with the published literature (13,14). The proteins that were most enriched in the capture with the double modification H3K9me3S10ph (bottom left, blue) showed very little overlap with the strongest binders of H3K9me3 or H3S10ph peptides. Atrx, which appears in this group, has been shown previously to bind to H3K9me3S10ph (15).

### Validation of quantification of peptide binding

**Figure 2.**
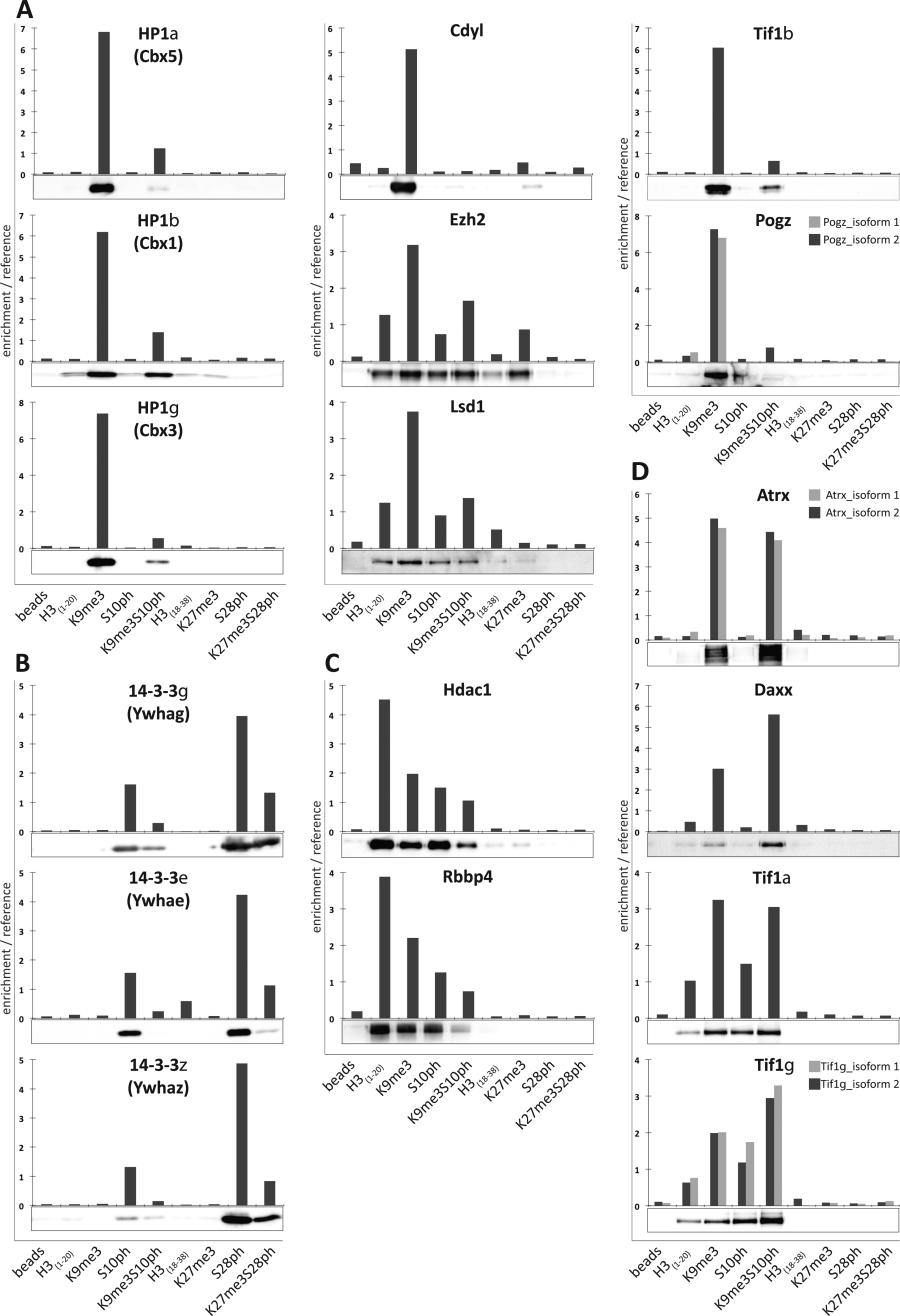
Quantification of peptide capture assays by western blotting closely corresponds to values obtained by mixed reference SILAC. Binding profiles of different groups of histone mark readers measured by mixed reference SILAC analysis of nuclear extracts from MPC11 (colum graphs) were compared with either quantitative western blot analysis of unlabelled extracts from the same cells (HP1 α, β and γ, Tif1 α, β and γ, 14**-**3-3ϵ and Atrx) or quantitative western blot analysis of tagged proteins expressed in HEK293T cells. The tagged constructs were HA-CDYL, Flag-Pogz, Daxx-myc-His, HDAC1-Flag, myc-Rbbp4, Flag-14-3-3γ and Flag-14-3-3ζ western blots are shown below the corresponding graphs. (**A**) H3K9me3 readers. (**B**) H3S10ph and H3S28ph readers. (**C**) Proteins that bind preferentially to unmodified H3_(1–20)_ peptide. (**D**) Top binders interacting with double modified peptide H3K9me3S10ph.

The ability of the super-SILAC based approach to accurately quantify protein binding to histone modifications was validated by analysing selected reader proteins for each peptide using quantitative western blotting and comparing the results with those obtained by SILAC (Figure 2, Supplementary Figure S3). For proteins for which antibodies suitable for western blotting were available, the analysis was carried out on extracts from MPC11 cells. Where suitable antibodies were not available, tagged versions of the proteins were transiently expressed in HEK293T cells The peptide capture was then performed on extracts from the transfected cells and the level of binding was quantified by western blotting using an antibody that recognized the tag. The results show that for a wide range of proteins, regardless of whether they were endogenous or transiently overexpressed, the binding profiles obtained by mixed reference SILAC in MPC11 cells corresponded closely with the results of quantitative western blots. These results confirm the accuracy of the quantification achieved using the super-SILAC based approach.

### Weighted correlation network analysis of the histone H3 tail interactome

Analysis of the data obtained from the nine duplicate experiments (eight peptide captures and the beads-only control), yielded a total of 1326 proteins identified by the Andromeda search engine at a FDR of 1%. We were intrigued by the fact that members of known complexes (FACT, PRC2, G9a/GLP) showed distinct and readily identifiable similarity of binding profiles and levels. This led us to consider whether the accurate quantification of binding using the super-SILAC based approach could allow us to generate binding ‘signatures’ that would allow high throughput identification of binding of novel complexes to specific combinations of modifications.

In order to identify groups of proteins with highly similar binding ‘signatures’, we adapted WGCNA (6) for analysis of proteomic peptide capture data. WGCNA is one of the most robust methods for construction of large networks in an unsupervised manner (16). It was developed to describe the correlation patterns among genes across microarray samples. Briefly, in WGCNA, a pair-wise Pearson correlation matrix is created for all expressed genes and the calculated correlations are then weighted using a power function to determine the connection strengths between any two genes in comparison to all other genes in the network. In the resulting gene network, co-regulated genes are grouped into modules, whose members share similar expression patterns across the entire dataset (6).

Although WCGNA was created to study gene expression networks, the scale-free topology network model it uses fits even better with protein–protein interaction networks. We decided therefore to use its ability to uncover tightly correlated modules within datasets to identify clusters of proteins with very similar binding ‘signatures’, potentially corresponding to histone reader complexes. WCGNA supports assembly of both signed and unsigned networks. However, since binding of members of multiprotein complexes to a histone mark as a single unit would lead to similar binding to each of the modifications for each complex member, we have limited our search to positive correlations only. For the analysis, we have selected proteins present in at least four measurements out of total 18 (*n* = 1023). Based on the strength of binding across the measured samples, we have calculated correlation coefficients for each of the proteins in the cohort. Next, we transformed the correlations into adjacencies with a power adjacency function. A power value of 15 was chosen as the soft threshold β. This was done in order to ensure the scale free topology of the resulting network, where most proteins (hubs) will be connected to only a few binding partners, and just a few hubs will be connected to a large number of other hubs.

**Figure 3.**
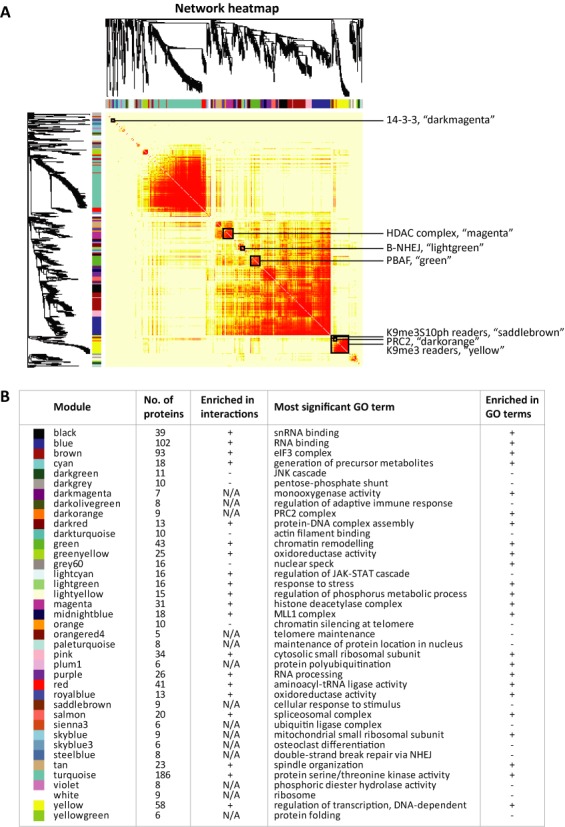
Weighted correlation network analysis (WGCNA) of the H3 tail interactome. A total of 1023 proteins, which were detected in at least 4 out of 18 pulldowns, were analysed by WGCNA based on their H/L ratios across the two sets of replicates. Proteins were clustered by their binding patterns as represented by the dendrogram and correlation heatmap. Clusters of proteins showing similar behaviour in the peptide capture assays are referred to as modules and denoted by colour. Proteins that could not be assigned to a module are labelled grey. (**A**) Heatmap of the topological overlap within the protein network. In the heatmap, each row and column corresponds to a protein. Intensity of red colouring indicates strength of the binding profile correlation (topological overlap) between pairs of proteins, with light colour corresponding to low topological overlap and progressively darker colours denoting higher topological overlap. Darker squares along the diagonal correspond to network modules. Selected modules, which are described in more detail in the text, are marked by black frames. (**B**) Table summarizing modules obtained by WGCNA. For each module, the assigned colour, name, number of proteins and the most significant GO term has been given. The modules significantly enriched in protein–protein interactions and GO terms in the STRING database are identified by ‘+’ in the relevant columns. ‘N/A’ denotes the modules comprising less than 10 proteins, for which enrichment in interactions could not be calculated.

Our analyses revealed that the H3 tail interactome does indeed show strong modularity (Figure 3A). In total, 39 non-overlapping modules with highly correlated proteins were detected, encompassing from five (set as the minimum module size) to 178 proteins. Modules were named after different colours according to the convention of WGCNA (6). Thirty-nine proteins were not assigned to any module, and were labelled with the colour grey. (Figure 3B and Supplementary Figure S4A, Supplementary Table S1).

To characterize the modules and the relationships between them, we have calculated the first principal component (eigenprotein) for each module (Supplementary Figure S4B and Supplementary Table S1). The eigenprotein corresponds to a theoretical ideal representative protein member among all proteins in the module. We have also calculated the connectivity values for each protein (Supplementary Table S1). The intramodular connectivity (kWithin) describes the correlation between the binding profile of a protein in the module and the module's eigenprotein, with the top hub proteins being characterized by the highest kWithin values.

The biological characteristics of identified modules were examined using existing data on protein–protein interactions that has been gathered in the publicly available STRING database. Of the 25 modules for which the number of proteins *n* ≥ 10 (minimum number of nodes required by STRING), 19 modules (76%) were significantly enriched in interactions (*p* ≤ 0.05), supporting the idea that the WGCNA modules can be used to identify complexes in the cells. Additionally, 24 out of a total of 39 modules (∼62%) and 20 out of 25 modules containing at least 10 proteins (80%) were significantly enriched in GO terms (Figure 3B and Supplementary Table S1).

### Characterization of modules that bind to specific combinations of histone modifications

**Figure 4.**
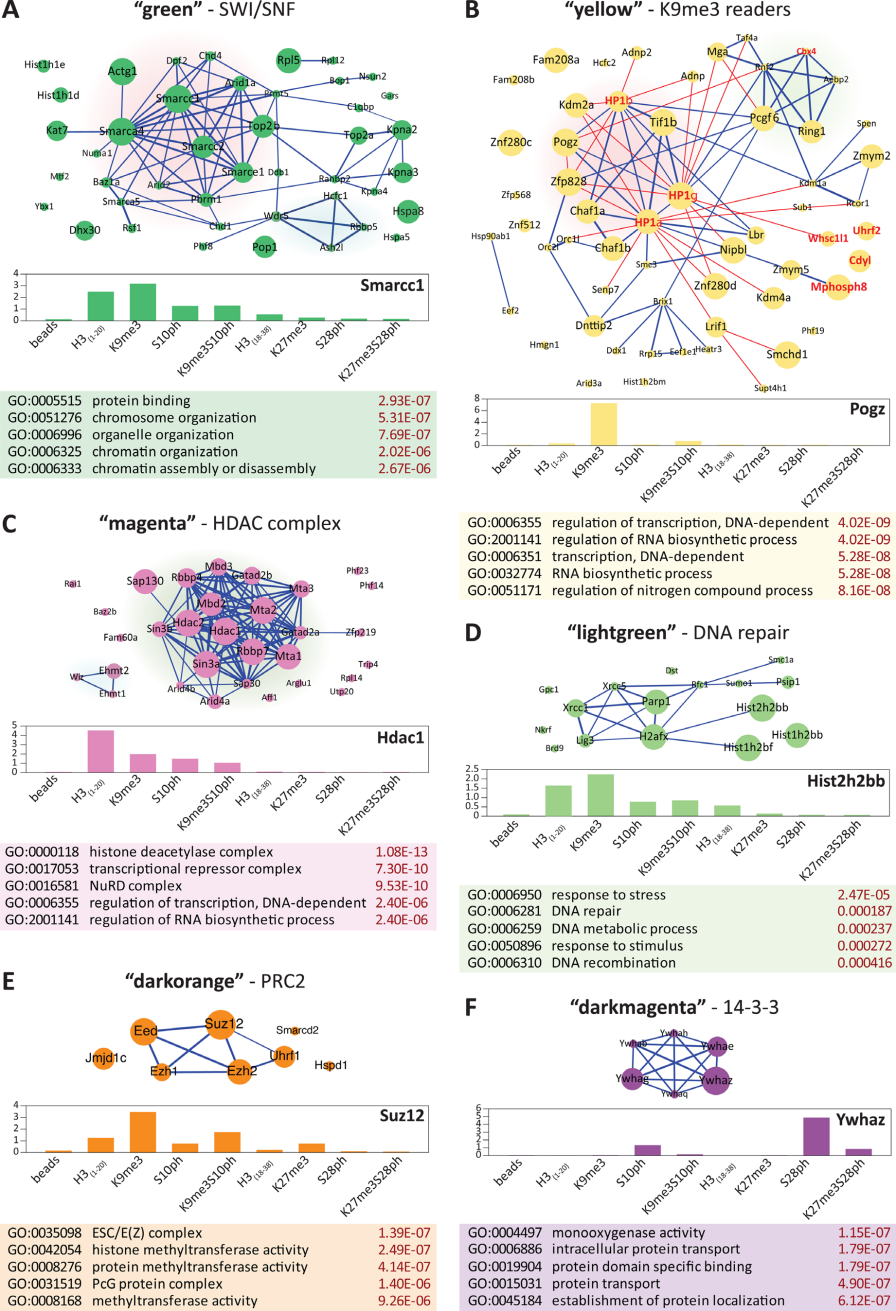
STRING analysis of protein–protein interactions in WGCNA modules. The network plots (upper panels) are based on the known and predicted interactions from the STRING database (version 9.1), with minimal confidence score of 0.4. The thickness of the blue lines representing interactions is proportional to the STRING confidence score. The node size corresponds to intramodular connectivity values (kWithin) in the WGCNA analysis. The bar graphs represent the binding profiles across all pulldowns for the top hub protein in the module (characterized by the highest intramodular connectivity values (kWithin)), with the y-axis representing the enrichment over the mixed reference for each pulldown (middle panels). Top five enriched GO terms in each module are given in the tables below. (**A**) The ‘green’ module. The cluster corresponding to the core SWI/SNF complex is highlighted in red. The Ash2l methyltransferase subcluster is highlighted in blue. (**B**) The ‘yellow’ module. HP1 family members and closely associated proteins are highlighted in red. The PRC1L4 complex members are highlighted in green. Additionally, the known direct trimethyl binders are labelled in red. The additional red lines denote interactions manually curated from the literature. (**C**) The ‘magenta’ module. The core HDAC complex is highlighted in green. The G9a/GLP complex is highlighted in blue. (**D**) B-NHEJ-containing ‘lightgreen’ module. (**E**) PRC2 complex clustered in the ‘darkorange’ module. (**F**) The ‘darkmagenta’ module composed of 14-3-3 family members.

A number of modules that were identified containing components of known protein complexes, as well as additional members not previously described, are shown in Figure 4. The network edges correspond to known protein–protein interactions; with the line width representing the combined confidence score from STRING database. The size of a node is correlated with intramodular connectivity (kWithin) from the WGCNA analysis, with the largest circles corresponding to the top hub proteins in the module.

Proteins that belong to a known SWI/SNF remodelling complex are grouped in the ‘green’ module, which binds to the unmodified and H3K9me3 modified H3 1–20 peptide (Figure 4A, Supplementary Figure S6 and Supplementary Table S1). The hub protein of the module, Smarcc1/Baf155 contains a SANT domain, which has been postulated to be a histone-binding module (17). It should be noted that the SANT domain of human SMARCC1 has an acidic surface, analogous to the SANT domain in the yeast Ada2 protein, which interacts primarily with unmodified histone tails (18). The four main ‘hub’ proteins in the module belong to the polybromo-associated BAF (PBAF) variant of the SWI/SNF complex. (19). Although binding of the PBAF chromatin-remodelling complex to H3K9me3 has not been described previously, one of its core proteins, the adenosine triphosphate-dependent helicase Smarca4/Brg1 directly interacts with Cbx5/HP1α. Interestingly, the residues in HP1α that are essential for this interaction were also shown to be critical for the silencing activity of Cbx5/HP1α (20). Members of the PBAF complex, including Smarca4/Brg1, Smarcb1/Baf47, Smarcc1/Baf155 and Smarce1/Baf57, are required for the repression of Nanog and other self-renewal genes upon mouse ESC differentiation (19). Moreover, knockdown of Smarcc1/Baf155 results in a block on formation of H3K9me3 foci during RA-induced differentiation of ES cells (19). These observations support the conclusions from our data that the ‘green’ module represents a histone H3 binding complex that binds to the histone H3 tail both with and without H3K9me3. Similar to a number of other H3 tail binders, phosphorylation of H3S10, either on its own, or in conjunction with H3K9me3, is sufficient to displace binding of the module.

Proteins that are characterized by strong binding to H3K9me3, which is almost completely abolished by simultaneous phosphorylation of S10, are clustered in the ‘yellow’ module (Supplementary Figure S6 and Supplementary Table S1). The members of this module also show very little binding to other peptides analysed in this study. The module is only moderately enriched in interactions according to STRING (Figure 4B, blue lines). However, detailed mining of the literature uncovered 24 additional protein–protein interactions within the module (Figure 4B, red lines), which were correctly predicted by WGCNA analysis (11,21–33). The core of the ‘yellow’ module consists of HP1 family members (Cbx1/HP1β, Cbx3/HP1γ and Cbx5/HP1α) and associated proteins, which are characterized by high kWithin values. Notably, Znf280c, despite being one of the predicted top hubs of the module was not identified as an interactor either by STRING or literature mining. However, Znf280c belongs to the ‘suppressor of hairy wing homolog’ family together with Pogz, Zfp828 and Znf280C, all of which are known HP1 binding proteins. This suggests that Znf280c is a good candidate for a novel HP1-dependent H3K9me3 reader protein. In addition to the HP1 family, the module contains a number of other known direct binders of H3K9me3 and includes several chromodomain-containing proteins (Cdyl, Adnp, Adnp2, Uhrf2, Mphosph8, Cbx4) (Figure 4B, labelled in red) (34–38). A subcluster corresponding to a PRC1-type polycomb complex was detected within the ‘yellow’ module, whereas no PRC2 proteins were present. The presence of Ring1, Ring2 and Pcgf6 in this complex and the association with Cbx3/HP1γ suggests that it may resemble the (PRC1)-like 4 (PRC1L4) complex described by Trojer *et al*. (39), which contains all four of these proteins.

The ‘magenta’ module (Figure 4C) is an example of a module that is highly enriched in interactions and provides a good validation of the ability of the method to detect binding of complexes to the histone tail. It contains proteins that belong to the well-characterized histone deacetylase/NuRD complex, with Hdac1, Hdac2 and Sin3a as the top hub nodes. The majority of known complex members are characterized by very high intramodular connectivity kWithin whereas the auxiliary proteins, most likely corresponding to non-specific contaminants, have low kWithin. The NuRD complex shows the highest level of binding to the unmodified histone H3 tail and a reduced level of binding to a tail that includes the H3K9me3 or H3S10ph modifications, with the lowest level of binding observed for the H3K9me3/S10ph combination (Supplementary Figure S6 and Supplementary Table S1). This binding could be mediated by the metastasis associated (MTA) family proteins (Mta1 and Mta2) within the NuRD complex, which has recently been shown to interact directly with the H3 tail via their C-terminal regions. Paralleling our results, the binding of MTA proteins was the strongest for unmodified H3 peptide, reduced in the presence of H3K9 trimethylation or acetylation and completely abolished by H3K4 methylation (40). Another factor that could be responsible for recruitment of the complex to histones is the Rbbp4 protein, which has recently been shown to bind to the unmodified H3 tail via its WD40 domain (41). A previous study also showed significant binding of Hdac1 to the unmodified tail of histone H3 and to a much lesser degree to H3K9me3, which fits well with our results (37).

The ‘lightgreen’ module (Figure 4D) closely resembles B-NHEJ, a ‘backup’ complex involved in the repair of double strand DNA breaks in the absence of the classical DNA–PK-containing D–NHEJ complex. In HeLa cells, this complex contains PARP1, DNA ligase III and XRCC1 as core proteins (42,43). It is noteworthy, that histone H1, identified as one of the top hubs in the module, has been postulated to be a critical stimulatory factor in this NHEJ pathway (44). The complex is very likely recruited to chromatin by PARP, which has been shown to interact directly with core histones, especially H3 and H4 (45). PARP1 is known to have important functions for the maintenance of heterochromatin (46), which is strongly enriched in the H3K9me3, mark and is not displaced from metaphase chromosomes (47), which are enriched for H3K9me3S10ph. This fits well with our data, which shows the strongest binding of the top hubs in this network to H3K9me3 and significant binding to H3K9me3S10ph (Supplementary Figure S6 and Supplementary Table S1).

Additional modules that are enriched in interactions according to STRING and contain members of known complexes are shown in Figure 4E and F and Supplementary Figure S5. They include the polycomb PRC2 complex (‘darkorange’ Figure 4E) and the 14-3-3 proteins (‘darkmagenta’, Figure 4F). Interestingly, the core of the ‘darkorange’ module, Ezh2, Suz12 and Eed showed ∼3-fold higher binding to H3K9me3 than to H3K27me3 (Figure 4E, Supplementary Figure S6 and Supplementary Table S1). Even though polycomb proteins are considered to be canonical readers of H3K27me3, there is also evidence of a role for H3K9me3 in polycomb recruitment. PRC2 proteins have been shown to bind to H3K9me3 through the WD40 domain of the Eed protein (48,49). Moreover, in our analysis, the polycomb PRC2 complex members Ezh2, Suz12 and Eed showed ∼3-fold higher binding to H3K9me3 than to H3K27me3 (Figure 4E). This agrees well with published data showing that the Kd for binding of Eed to an H3K9me3 peptide is around three to four times lower than for H3K27me3 (see Supplementary Table S1 in Margueron *et* *al*. (48) and Figure 1 in Xu *et* *al*. (49)) and supports the idea that H3K9me3 may have a more important role in mediating polycomb binding than was previously thought (3,50). Notably, the auxiliary protein Uhrf1 found in the ‘darkorange’ module has previously been shown to interact with PRC2 members Ezh2 and Suz12 in prostate cancer cells, where elevated UHRF1 levels correlate positively with an increase in EZH2 and were associated with poor clinical outcome (51).

All of the 14-3-3 family members, which constituted the major group of phospho mark readers in our study (Figure 4F) cluster together in the ‘darkmagenta’ module. Although studies on the role of the 14-3-3 proteins have focused on their role in recognizing H3S10ph at the promoters of transcriptionally active genes, our experiments showed that they bind much more efficiently to H3S28ph than to H3S10ph. This result is in agreement with studies that directly examined the *K*_d_ of 14-3-3 proteins for H3S10ph and H3S28ph (13,14). Phosphorylation of histone H3 at S10 and S28 has been implicated in transcriptional activation (52). Our results indicate that H3S28ph is likely to be a major recruiter of 14-3-3 proteins to active gene promoters (Supplementary Figure S6 and Supplementary Table S1).

### Identification of a novel complex centred on Atrx that binds to the H3K9me3/S10ph double modification

**Figure 5.**
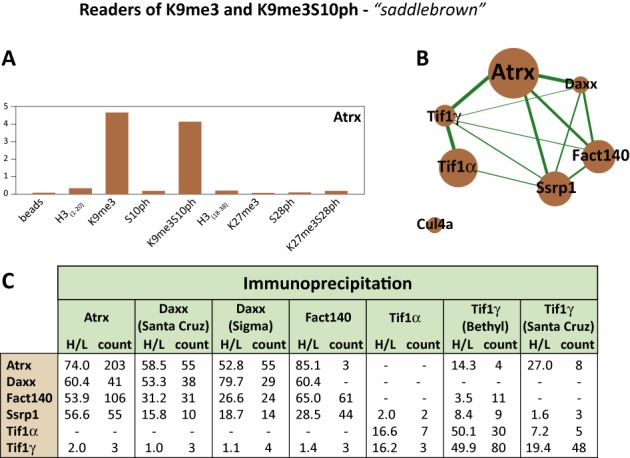
Antibody pulldowns confirm interactions between the members of the ‘saddlebrown’ Atrx-Daxx-FACT module. Nuclear lysates from SILAC-labelled MPC11 cells were subjected to IP with the indicated antibodies that recognize the members of the ‘saddlebrown’ module (‘heavy’-labelled extracts) or with isotypic non-specific immunoglobulin (light-labelled extracts). The ‘heavy’ pulldowns were then mixed 1:1 with their relevant ‘light’ isotype controls and the ‘heavy-to-light’ (H/L) ratios for proteins present in the pulldowns were determined by mass spectrometry. (**A**) The binding profile across the nine peptide pulldowns for the hub protein Atrx. The y-axis represents enrichment over the mixed reference. (**B**) Interaction network based on IP experiments in MPC11 cells. The thickness of the interaction lines (in green) is proportional to SILAC ‘H/L’ ratios representing the enrichment in the specific IP normalized against the bait pulldown. The node size corresponds to intramodular connectivity values (kWithin) in the WGCNA analysis. (**C**) Summary of the H/L ratios and corresponding ratio counts obtained for members of the ‘saddlebrown’ module in the SILAC-IP experiments. Each column lists the proteins pulled down with the indicated antibody.

We next asked whether we could use the predictive qualities of the analysis to uncover novel chromatin reader complexes. We chose the ‘saddlebrown’ module, whose members bind to H3K9me3, but are not displaced by S10 phosphorylation and which contains known chromatin-associated proteins (Figure 5A). To verify whether the proteins indeed interacted together in MPC11 cells as suggested by the results of WGCNA analysis, we performed a series of IP assays targeting five proteins of the ‘saddlebrown’ module using nuclear extracts from heavy-labelled MPC11 cells. The proteins that were immunoprecipitated were Atrx (the ‘top hub’ protein for this module), Daxx, FACT complex member Fact140, Tif1α and Tif1γ. As the negative control, IPs with corresponding isotype control antibodies were performed in light-label format. Specific ‘heavy’ pulldowns were then mixed in a 1:1 ratio with their respective ‘light’ negative controls. H/L SILAC ratios, corresponding to the enrichment over the negative control were measured by mass spectrometry. The values obtained were used to construct an interaction network in Cytoscape v. 3.1.0 (9) (Figure 5B). The width of the lines is proportional to enrichment/negative control normalized against the ‘bait’ pulldown, whereas the size of the nodes shows intramodular connectivity kWithin value of the protein from the WGCNA analysis. The results of the pulldown assays, including H/L SILAC ratios and the ratio count are summarized in the table below the module network (Figure 5C).

As predicted by our analyses, all ‘saddlebrown’ module proteins, apart from Cul4a formed an interaction network in our IP experiments. The WGCNA-derived ‘hub protein’ for this module, Atrx, had the strongest connections with the rest of the module in the IP analysis, confirming the utility of WGCNA analysis of peptide capture data for identifying novel histone binding complexes. It is notable that culin 4a, which was not detected at all in our IP experiments, was assigned the lowest kWithin value among the ‘saddlebrown’ module proteins. Moreover, we uncovered strong interactions between Atrx and both of the FACT subunits. These proteins show similar binding patterns, with comparable binding to H3K9me3 and H3K9me3S10ph marks and no binding to H3S10ph alone (Figure 5A, Supplementary Figure S6 and Supplementary Table S1).

## DISCUSSION

A key driver of epigenetic effects is the interaction of the soluble nuclear proteome with chromatin. According to current thinking, much of the information content generated by this interaction is provided by specific binding of reader proteins to different histone modifications. This can occur through classic ‘lock and key’ recognition of a single histone modification, but the binding can also be affected by the presence of multiple histone modifications that alter the charge environment of the region and either cause steric hindrance of binding or generate new binding epitopes. A further complication is the fact that components of the nuclear proteome interact with one another to form large multi-protein complexes, the composition of which can vary in response to short-term signalling and longer-term development and cell differentiation.

This paper describes a novel high-throughput approach for identifying protein complexes that bind to different combinations of histone modifications. We have used this approach to carry out a systematic analysis of the interactome of the repressive histone marks H3K9me3 and H3K27me3 and the interplay between these trimethyl marks and the phosphorylation at their neighbouring lysines, H3S10 and H3S28. The strategy makes use of an adapted super-SILAC method for high-throughput, accurate quantification of binding of nuclear proteins to different combinations of modifications. This method is easily scalable and relatively simple, which makes it feasible for laboratories with access to standard mass spectrometry equipment to use it. Moreover, by using heavy-labelled cell line(s) to generate the mixed reference, this approach can easily be adapted to analyse a wide range of samples that are hard to label, including primary cells and purified cell populations from different stages of the cell cycle. In addition to the results described in this paper, we have also successfully used this approach to characterize histone mark readers in primary activated B cells (data not shown).

We have coupled the super-SILAC approach with the use of WGCNA analysis of binding profiles to uncover the modular organization of chromatin-binding proteins. A critical factor in the construction of the WGCNA network from our dataset was the quality of the protein quantification achieved through use of the mixed super-SILAC reference. WCGNA was originally created to study gene expression networks, but it has since been successfully adapted for the study of tomato metabolomes (53), for clustering breast cancer patients into groups with distinct prognostic outcomes based on immunohistochemical staining (54) and for analysing the protein interactome for mouse huntingtin across different brain regions and ages (10). Our study further extends the usefulness of WGCNA to the characterization of epigenetic reader complexes. WGCNA helps to reduce the complexity of large, multivariant datasets by clustering proteins into modules, thereby facilitating the identification of biologically relevant clusters. By combining the protein modules identified by WGCNA with published interaction data from the STRING database, it becomes possible to construct interaction networks that contain putative complexes with specific histone modification binding profiles.

Our analysis of the interactomes of H3K9me3 and H3K27me3 and their corresponding K9me3/S10ph and K27me3/S28ph methyl-phospho double modifications, together with a comparison with binding to the unmodified histone tail, has provided a number of insights into the dynamics of binding of protein complexes to histone H3 modifications. As expected, the analysis identified several known interaction modules for proteins that bind to H3K9me3 and H3K27me3. The largest of these is centred on the HP1 proteins and is almost completely displaced from the H3 tail by the presence of S10ph. This cluster is likely to encompass several known complexes containing different HP1 isoforms. Of particular interest is the clustering of the components of the (PRC1)-like 4 (PRC1L4) complex within the ‘yellow’ module. Significantly, the PRC1L4 cluster identified as H3K9me3 binders in our analysis lacks the L3mbtl2 protein, which has been proposed to be a component of PRC1L4. This correlates with the results of Trojer *et al*. (39) who showed that the L3mbtl2-containing PRC1L4 complex binds only to regions that lack H3K9me3 and H3K27me3. Our results provide evidence of the existence of a variant PRC1L4 complex that binds to H3K9me3, probably through interaction with Cbx3/HP1, and has H2A mono-ubiquitinating activity (inferred from the presence of RNF1 and RNF2) while lacking L3mbtl2 and associated H3K9 KMTs.

Our results also identify strong binding of members of the PRC2 complex to H3K9me3. The region containing H3K27me3 is relatively barren of interactions and shows weaker binding of the PRC2 polycomb complex than H3K9me3. Although this goes against the canonical view of polycomb binding specificity, our results agree with the findings of two separate *in vitro* studies (48,49). The exception to the low binding to H3_(18–30)_ region is the avid binding to H3S28ph exhibited by the 14-3-3 proteins. The absence of other interactors from this module supports the idea that the 14-3-3 proteins, which bind predominantly as dimers (55,56) act as transient adaptors for a large number of different proteins. In addition, we have identified a novel binding capability for a known Swi/Snf complex (PBAF) for the unmodified and H3K9me3-modified aa1–20 region of the histone H3 tail, which is displaced by S10 phosphorylation.

A critical test of the approach that we have used is whether it can identify previously undescribed complexes. Using the WGCNA clustering of binding profiles, we have obtained evidence for the existence of a novel complex that includes Atrx and the histone chaperone Daxx, Tif1γ and Tif1α and the FACT complex members Fact140 and Ssrp1. The existence of the complex was corroborated by a separate IP analysis, which showed that all six proteins interact. A seventh candidate member of the complex, Cul4a, was excluded by the IP analysis. Analysis of *in vitro* binding to modified peptides has shown that phosphorylation of H3S10 does not result in any reduction of binding of Atrx to H3K9me3 (15). This is due to the fact that binding of Atrx to H3K9me3 occurs through an atypical composite H3K9me3-binding pocket, distinct from the conventional trimethyl-lysine-binding aromatic cage (21). The positioning of the histone tail in this pocket allows the Atrx ADD domain to bind independently of the S10 and K14 modification status of the H3 histone tail (15). This finding, together with the results of our analysis, which place the Atrx at the hub of both peptide pulldown and IP derived interaction networks, strongly suggests that Atrx is the major factor responsible for the recruitment of the ‘saddlebrown’ complex to chromatin and a *bona fide* reader of the double phospho-methyl mark. This conclusion is supported by the observation that Atrx is retained at pericentric heterochromatin in G2/M cells (57) despite the fact that HP1 binding is displaced from H3K9me3 during G2/M due to phosphorylation of the adjacent S10 (58). At least one member of the FACT complex, Ssrp1, has been reported to interact with methylated H3K9 (59). Our data clearly show that binding of Ssrp1 and Fact140 to H3K9me3S10ph is almost identical to the binding observed for H3K9me3 only. Tif1γ has a PHD domain that also lacks the trimethyl-lysine-binding aromatic cage (60) and can therefore potentially bind H3K9me3/S10ph. The presence of both of these non-canonical H3K9me3-binding proteins in the complex could facilitate spreading of the complex on H3K9me3-enriched heterochromatin and retention through mitosis.

Atrx and Daxx are known to interact with one another and to be involved in H3.3 deposition at centromeres and telomeres (57,61–64), and Atrx has been shown to be important for chromosome stability in mouse oocytes and early embryos (61). The FACT complex members Ssrp1 and Fact140 have also been found to be associated with centromeres and have been implicated in deposition of CENP-A (65). Our analysis provides evidence of cooperation between Atrx/Daxx and the FACT subunits and suggests that they form a complex that can be targeted to centromeric regions by binding to H3K9me3/S10ph, which are highly enriched in these regions.

In summary, we have described a high throughput approach that makes it possible to compare binding of protein complexes that recognize specific combinations of histone modifications. An important feature of the WGCNA analysis is that the resolution of the method for detecting binding of complexes is actually enhanced by increasing the number of peptides that are compared. This makes the approach an ideal one for constructing binding maps that compare binding to large numbers of different combinations of modifications. Construction of these types of maps using binding to peptides or reconstituted nucleosomes will be important for understanding how the histone code is translated into specific biological readouts.

The mass spectrometry proteomics data have been deposited with the ProteomeXchange Consortium (66) via the PRIDE partner repository with the dataset identifier PXD001273.

## SUPPLEMENTARY DATA

Supplementary Data are available at NAR Online.

SUPPLEMENTARY DATA

## ACCESSION NUMBER

The proteomic data has been submitted to PRIDE database, under the project name: Histone H3 tail interactome in MPC11 murine myeloma cells. Project accession: PXD001273.
