# Supplementary material for: Identification of protein complexes that bind to histone H3 combinatorial modifications using super-SILAC and weighted correlation network analysis
Source: Nucleic Acids Res. 2015 Jan 20;43(3):1418–32. doi: 10.1093/nar/gku1350 (PMC4330348; doi:10.1093/nar/gku1350)
Supplement: SUPPLEMENTARY DATA [file supp_43_3_1418__index.html]

Identification of protein complexes that bind to histone H3 combinatorial modifications using super-SILAC and weighted correlation network analysis — SUPPLEMENTARY DATA 

# Identification of protein complexes that bind to histone H3 combinatorial modifications using super-SILAC and weighted correlation network analysis

## SUPPLEMENTARY DATA

**Files in this Data Supplement:**

- Supplementary Figures
- Supplementary Tables
