# Supplementary material for: Identification of protein complexes that bind to histone H3 combinatorial modifications using super-SILAC and weighted correlation network analysis
Source: Nucleic Acids Res. 2015 Jan 20;43(3):1418–32. doi: 10.1093/nar/gku1350 (PMC4330348; doi:10.1093/nar/gku1350)
Supplement: SUPPLEMENTARY DATA [file supp_gku1350_nar-02443-m-2014-File007.pdf]

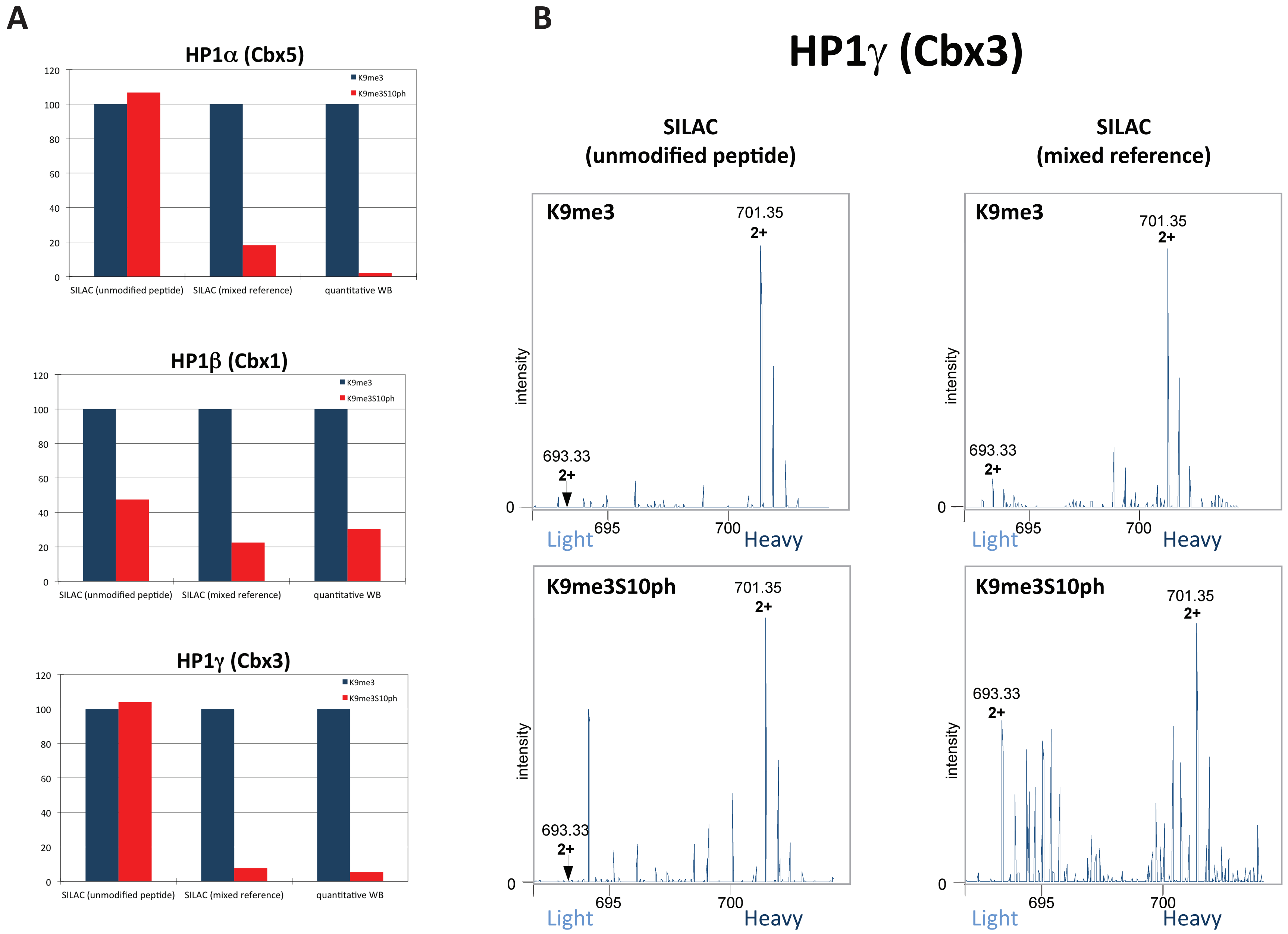

Supplementary Figure 1. Quantification of the effect of S10-phosphorylation on the binding of HP1α to the $H3_{(1-20)}$K9me3 peptide using pulldown with the unmodified peptide or mixed pulldowns as the internal reference. (A) Comparison of quantification of HP1 family members from MPC11 nuclear extract binding to H3K9me3 versus H3K9me3/S10ph by SILAC using pulldown with the unmodified peptide as the reference, super-SILAC type approach with mixed reference and quantitative western blot (WB). In the case of 2 out of 3 HP1 family members, SILAC in conjunction with unmodified peptide pullown as the reference failed to show any significant displacement from H3K9me3 upon phosphorylation of S10. (B) Mass spectra of an HP1γ peptide obtained with SILAC with unmodified peptide (left) and mixed reference (right). The arrow indicates the absence of signal in the light reference sample used in the first approach.

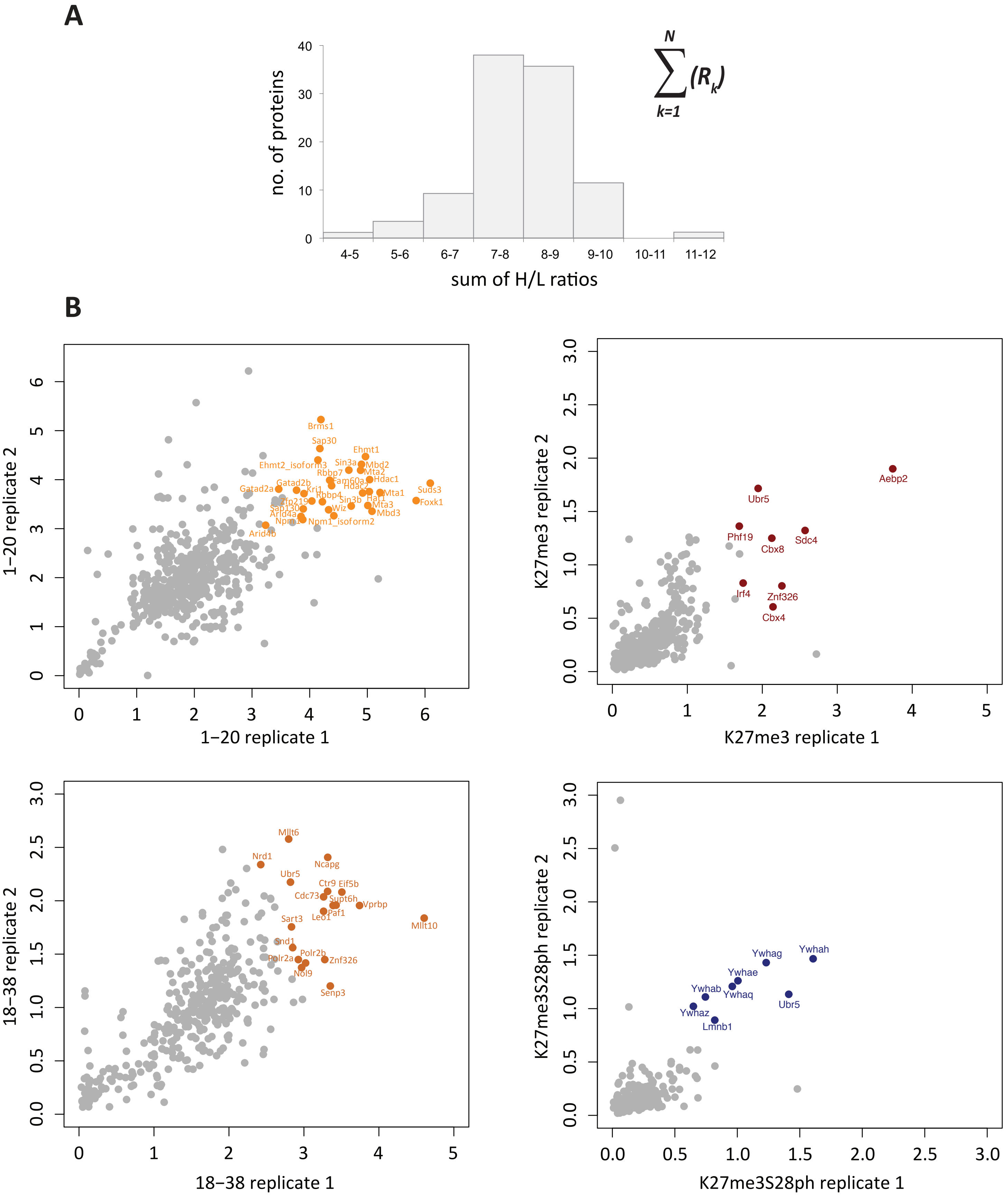

Supplementary Figure 2. Mixed reference SILAC analysis of proteins pulled down with modified peptides spanning H3 aa 1-38. (A) Protein frequency histogram of the sums of the SILAC "heavy-to-light" (H/L) ratios across the 9 pulldowns. Each histogram bar represents the proteins for which the sum of the pulldown/reference ratios falls within the interval indicated on the x-axis on either side of the bar. Only proteins that had no missing values were included in the calculation. For each protein, the sum of the ratios R over k = 1,2...N pulldowns should approximate N. (N corresponds to 9 in this case) (B) Scatter plots of replicate 1 vs. replicate 2 for $H3_{(1\text{-}20)}$ unmodified peptide pulldown (top left), $H3_{(18\text{-}38)}$ unmodified peptide pulldown (bottom left), H3K27me3 (top right) and H3K27me3S28ph (bottom right). The x and y axes represent the H/L ratios, which correspond to enrichment over the mixed reference. The top binders for each peptide have been highlighted in orange (for $H3_{(1\text{-}20)}$ unmodified), dark orange ($H3_{(18\text{-}38)}$ unmodified), dark red (K27me3) and dark blue (H3K27me3S28ph).

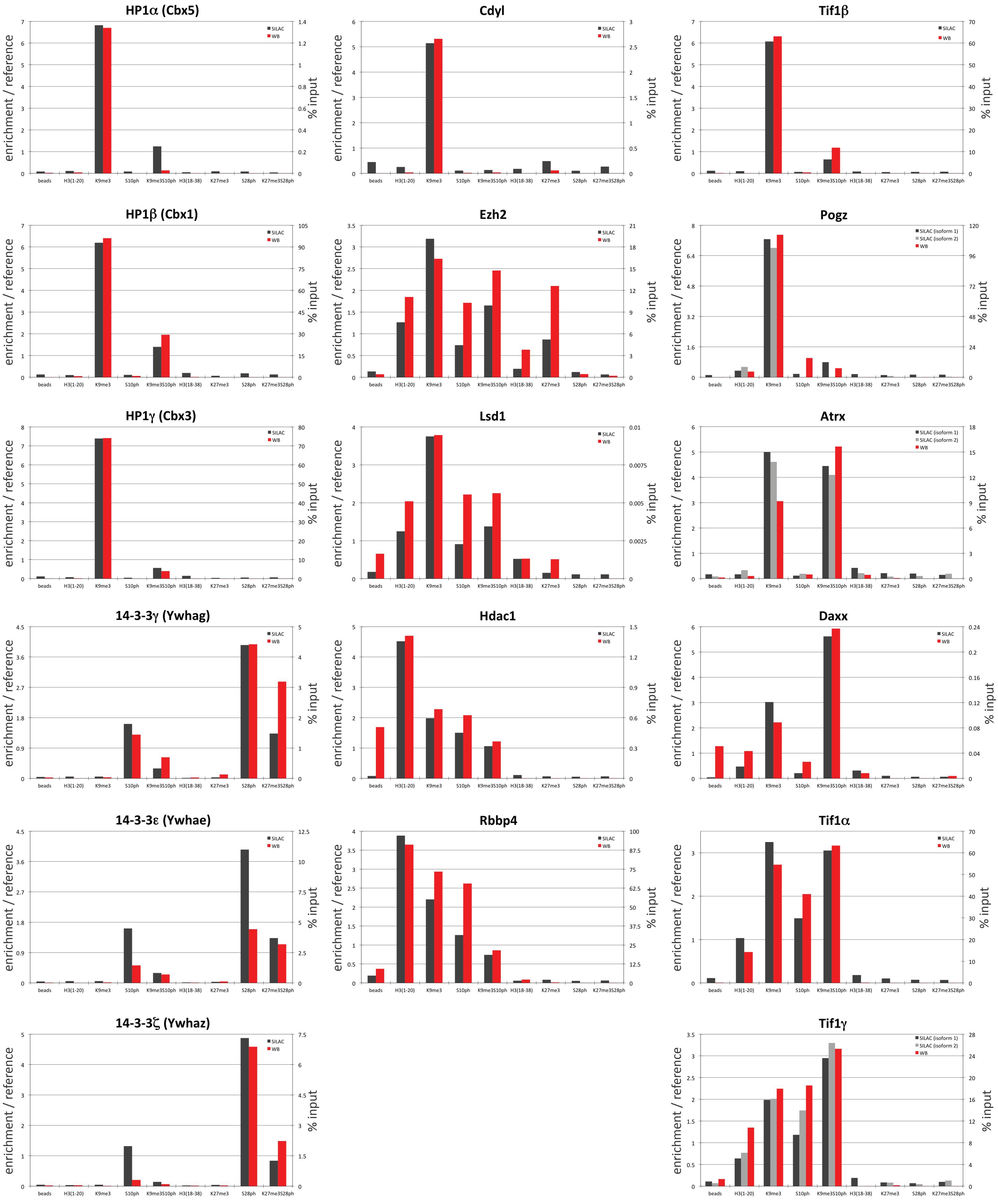

Supplementary Figure 3. Quantification of peptide capture assays by quantitative western blotting closely corresponds to values obtained by mixed reference SILAC. Binding profiles of different groups of histone mark readers measured by mixed reference SILAC analysis of nuclear extracts from MPC11 were compared with either quantitative western blot analysis of unalbelled extracts from the same cells (HP1α, HP1β and HP1γ, Tif1α, Tif1β and Tif1γ, 14-3-3ε and Atrx) or quantitative western blot analysis of tagged proteins expressed in HEK293T cells. The tagged constructs were HA-CDYL, Flag-Pogz, Daxx-myc-His, HDAC1-Flag, myc-Rbbp4, Flag-14-3-3γ and Flag-14-3-3ζ. SILAC quantification is represented by black and grey bars plotted on the left Y axis; western blot quantification is plotted in red on right Y axis.

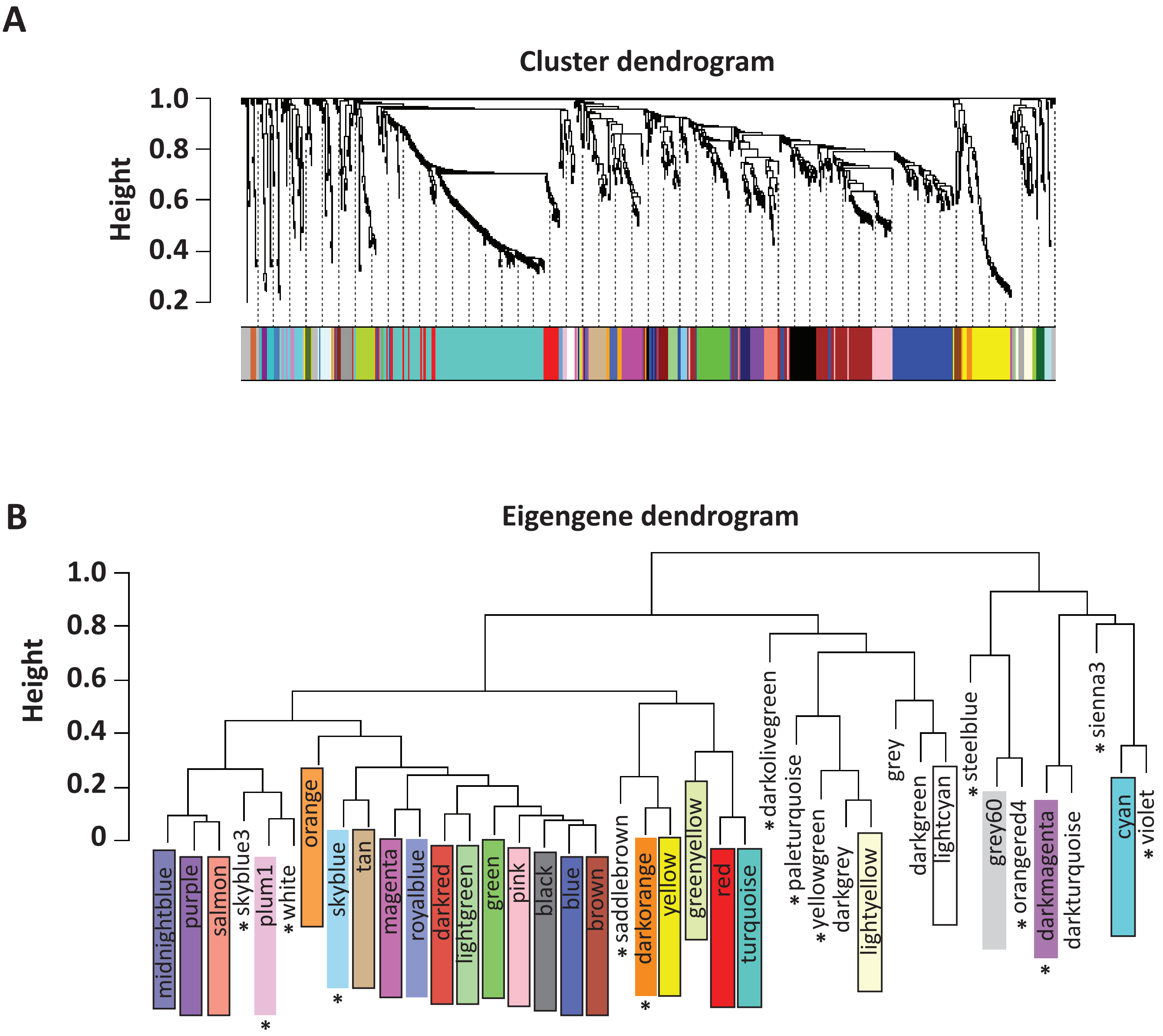

Supplementary Figure 4. WGCNA cluster and eigengene dendrograms. (A) Cluster dendrogram generated by hierarchical clustering of proteins on the basis of topological overlap. Each branch represents a single protein. The horizontal bar beneath the dengrogram indicates modules of correlated proteins. (B) Hierarchical clustering of module eigenproteins that summarize the modules found in the clustering analysis. Branches of the dendrogram group positively correlated eigenproteins together. Eigenproteins of modules significantly enriched in protein-protein interactions (p ≤ 0.05) in the STRING database are shown as boxed. Significant enrichment in GO terms is denoted by highlighting. The asterisks indicate modules comprising less than 10 proteins, for which the enrichment in interactions could not be calculated. Notably, the 3 modules with high affinity for K9-trimethylated peptides (HP1/“yellow”, PRC2 complex/“darkorange” and K9me3S10ph-binders/“saddlebrown”) cluster together.

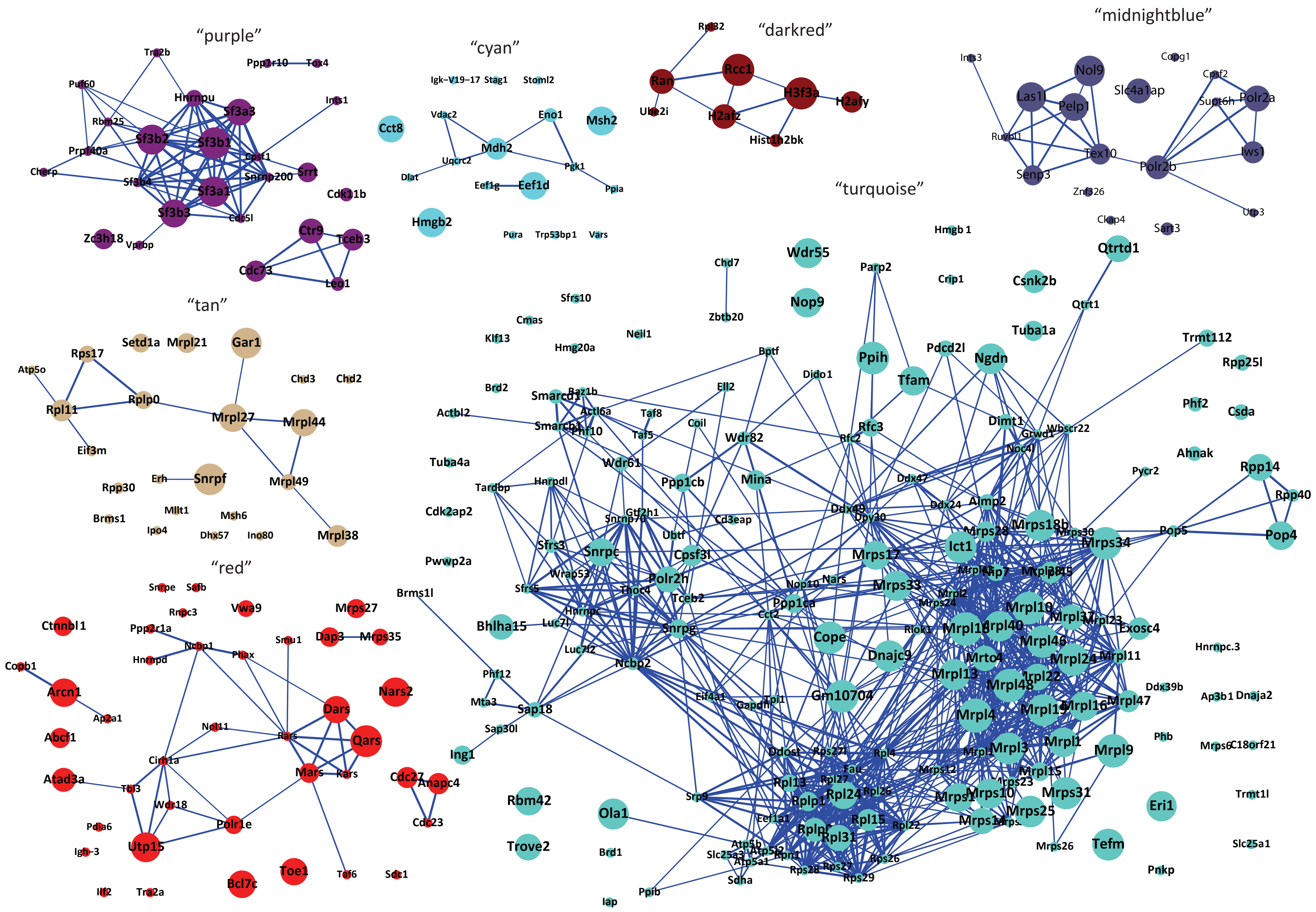

Supplementary Figure 5.

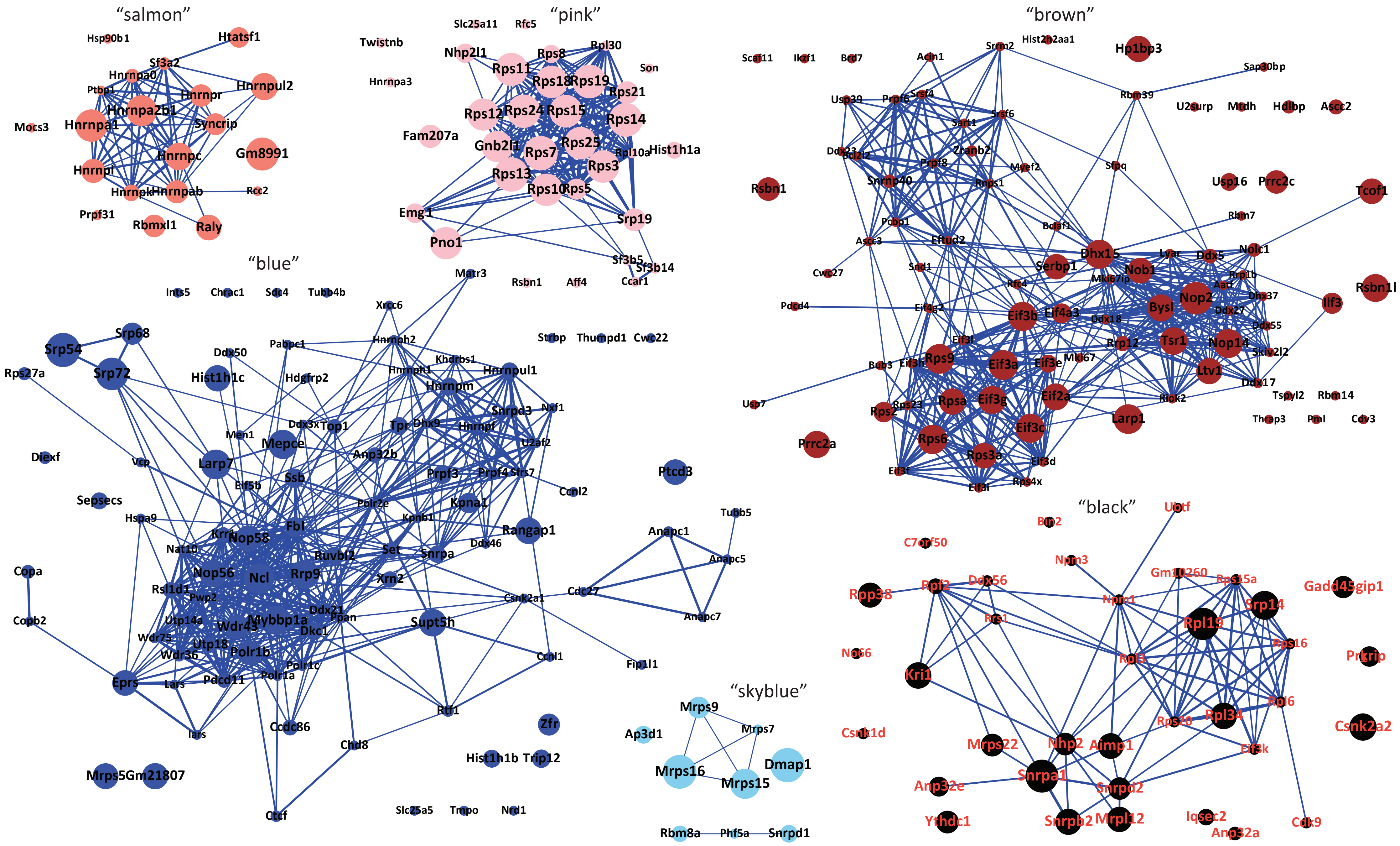

Supplementary Figure 5. Network plots of protein-protein interactions in WGCNA modules that are enriched in interactions according to the STRING database (version 9.1). The minimal confidence score for an interaction to be included was set at 0.4. The thickness of the edges (blue), representing interactions, is proportional to the STRING confidence score. The node size corresponds to intramodular connectivity values (kWithin) in the WGCNA analysis. The density of the interactions between proteins with similar binding profiles provides a good demonstration of the ability of WGCNA to detect complexes

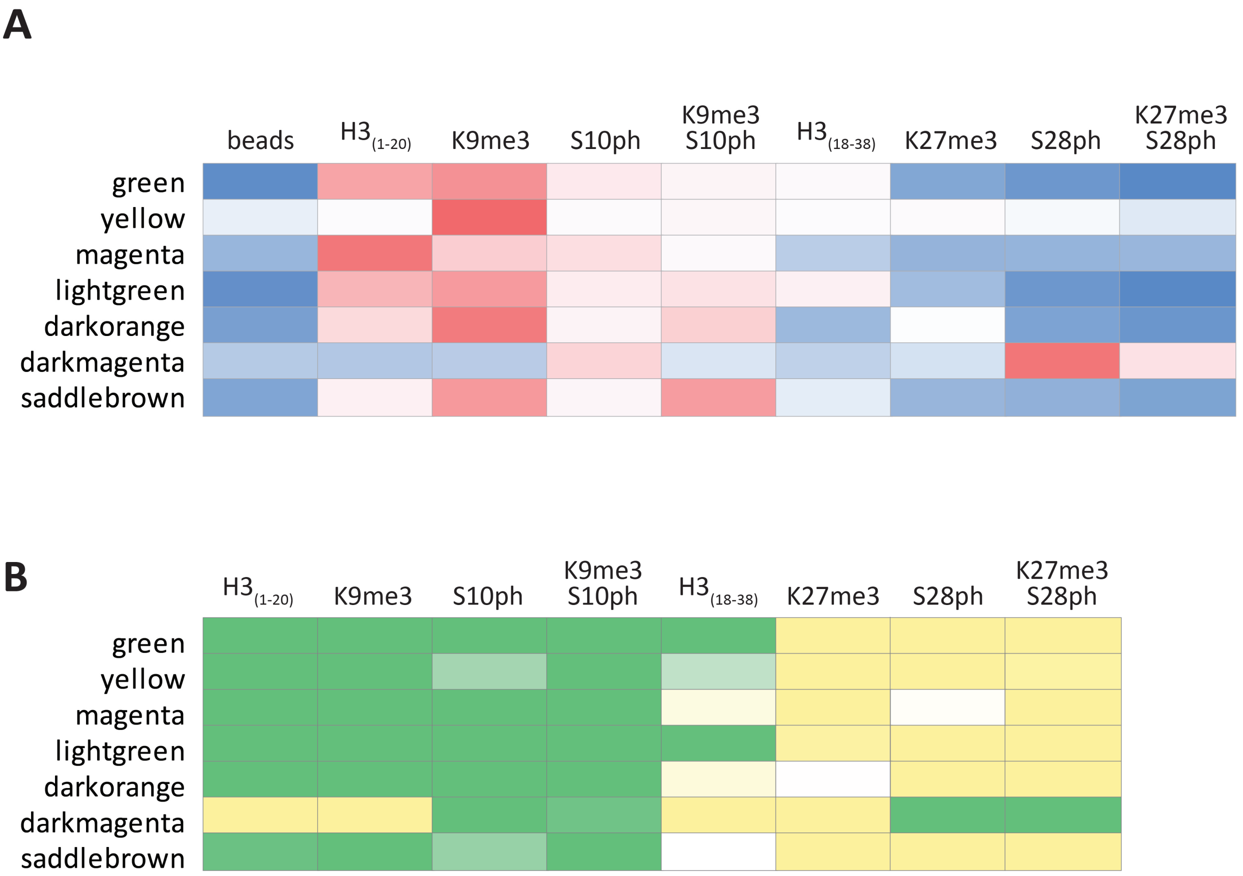

Supplementary Figure 6. Summary of the H3 tail binding for the major WGCNA modules. (A) Heatmap representing the strength of binding of the module to indicated peptides. The weak binding is indicated by the colour blue (the darker the colour, the less binding is observed). The enriched binding is indicated by the colour red, with the intensity of the colour corresponding the strength of the enrichment. (B) Heatmap representing the significance of enrichment on specific peptides in comparison to "bead-only" negative control for each module. Green denotes significant binding, whereas yellow denotes lack of significant enrichment compared to the negative control. The corresponding p values can be found in Supplementary Table 1.

Rationale for using WGCNA to characterise complexes that bind to combinatorial histone modifications

Analysing of binding of complexes to combinatorial histone modifications presents particular challenges. Standard analyses of biological datasets typically begin by identifying discrete components (for example, individual genes or proteins that are differentially expressed between 2 experimental conditions). These components are compared individually. This leads to loss of information as it does not reflect the continuous nature of the underlying co-expression patterns and only considers highly dissimilar components. An additional problem is that classical testing methods are powerful only when investigating small numbers of variables at a time. Increasing the number of components, which is what happens with combinations of a number of histone modifications, results in a very large number of permutations that would be impossible to analyse. Network analyses make it possible to characterize datasets with large numbers of components by identifying nodes of interest and also studying the properties of the network as a whole. This enables identification of highly connected modules and the relationships between them as well as their associations with external traits.

WGCNA is an efficient analysis method, which reduces the dimensionality of large system biology datasets from tens of thousands of genes or proteins to a handful of modules comprised of nodes that share similar expression/binding pattern and, potentially, related biological functions. Clustering proteins with similar binding profiles can shed light on nodes with unknown function by associating them with nodes that have already been well annotated. Moreover, WGCNA makes it possible to identify meaningful modules by testing their enrichment in known biological categories (eg. GO terms). WGCNA assumes 'scale-free' topology of the networks, which means that their connectivity distributions follow an inverse power law; ie. the networks have a small number of highly connected nodes while the rest of the nodes display low connectivity. This assumption is particularly relevant in the context of protein-protein interaction networks (1) that underlie the formation and binding of protein complexes to histone modifications. An additional advantage of WGCNA is that it provides a quantitative measure of module membership, which makes it possible to quantify how similar the binding pattern of a protein is to the overall binding profiles of other members of the same

module, and to identify proteins that are promising candidates for direct binders of histone modifications. These binders would drive the binding of other members of the module and will therefore tend to appear among the proteins with the highest module membership.

1. Barabasi, A.L. (2009) Scale-free networks: a decade and beyond. *Science*, **325**, 412-413.
